# Supplementary material for: Spatial heterogeneity of menstrual discriminatory practices against Nepalese women: A population-based study using the 2022 Demographic and Health Survey
Source: PLOS Glob Public Health. 2024 Nov 13;4(11):e0003145. doi: 10.1371/journal.pgph.0003145 (PMC11560001; doi:10.1371/journal.pgph.0003145)
Supplement: S1 Table — (DOCX) [file pgph.0003145.s001.docx]

**S1 Table:** Factors associated with menstrual taboos/restrictions

| **Predictor/**  **control variable** | **Variable type as recorded in 2022 NDHS** | **Data used in deriving the variable** | **Variable used in the study** | **Categories/**  **groups** |
| --- | --- | --- | --- | --- |
| Age | Continuous and Categorical | Individual data | Age in years of the respondent categorized into 7 groups. | 15-19, 20-24, 25-29, 30-34, 35-39, 40-44, 45-49 |
| Education level | Categorical | Individual data | Highest level of education of the survey respondent categorized into 4 groups | None, Basic, Secondary, Higher |
| Caste/Ethnicity | Categorical | Individual data | Respondent’s ethnicity grouped into 6 categories. Janajati (hill janajati, terai janajati, newar), Madhesi (terai brahmin/chhetri, other terai caste), Dalit(hill dalit, terai dalit), Brahmin/Chhetri(hill Brahmin, hill chhetri), Muslim, Others | Janajati, Madhesi, Dalit, Brahmin/Chhetri,  Muslim, Others. |
| Region | Categorical | Individual data | The seven first level administrative regions (provinces) of Nepal where the respondent resided at the time of the survey. | Bagmati, Gandaki, Karnali, Koshi, Lumbini, Madhesh, Sudurpashchim |
| Gender of the household head | Categorical | Individual data | Gender of the household head as reported by the respondent. | Male, Female |
| Age of the household head | Continuous | Individual data | Age of the household head as reported by the respondent. It was categorized into 6 groups | 15-24, 25-34, 35-44, 45-54, 55-64, 65 and over |
| Residence | Categorical | Individual data | Current area of residence whether urban(metropolitan, sub-metropolitan or municipality) or rural | Rural, urban |
| Wealth index | Categorical | Household data | In DHS surveys, household characteristics used to assess wealth are assigned factor scores using the principal components analysis (PCA). Using the first principal component, the sample is divided into quintiles, each with an equal number of households. Key characteristics included in this analysis were: drinking water source, toilet type, shared facilities, materials for the floor, walls, and roof, cooking fuel, household services, and possessions such as electricity, TV, vehicles, land size, livestock, bank accounts, and window types. | Poorest, poorer, middle, richer, richest. |
