# Supplementary material for: Spatial heterogeneity of menstrual discriminatory practices against Nepalese women: A population-based study using the 2022 Demographic and Health Survey
Source: PLOS Glob Public Health. 2024 Nov 13;4(11):e0003145. doi: 10.1371/journal.pgph.0003145 (PMC11560001; doi:10.1371/journal.pgph.0003145)
Supplement: S2 Table — (DOCX) [file pgph.0003145.s002.docx]

**S2 Table:** Social-demographic and economic characteristics of survey participants

|  | | **Total(N=14845)** | |
| --- | --- | --- | --- |
| **Variable** | **Category** | **Number(n)** | **Proportion (%)** |
| Age | 15-19 | 2,643 | 17.8 |
|  | 20-24 | 2,637 | 17.8 |
|  | 25-29 | 2,435 | 16.4 |
|  | 30-34 | 2,144 | 14.4 |
|  | 35-39 | 2,025 | 13.6 |
|  | 40-44 | 1,629 | 11.0 |
|  | 45-49 | 1,332 | 9.0 |
| Education | Basic | 4,595 | 31.0 |
|  | Higher | 656 | 4.4 |
|  | No education | 3,796 | 25.6 |
|  | Secondary | 5,798 | 39.1 |
| Region | Bagmati | 3,062 | 20.6 |
|  | Gandaki | 1,401 | 9.4 |
|  | Karnali | 909 | 6.1 |
|  | Koshi | 2,493 | 16.8 |
|  | Lumbini | 2,691 | 18.1 |
|  | Madhesh | 3,010 | 20.3 |
|  | Sudurpashchim | 1,279 | 8.6 |
| Religion | Buddhist | 970 | 6.5 |
|  | Christian | 445 | 3.0 |
|  | Hindu | 12,374 | 83.4 |
|  | Kirat | 365 | 2.5 |
|  | Muslim | 682 | 4.6 |
|  | Other | 8 | 0.1 |
| Residence | Rural | 4,667 | 31.4 |
|  | Urban | 10,178 | 68.6 |
| Ethnicity | Brahmin/Chhetri | 4,152 | 28.0 |
|  | Dalit | 2,240 | 15.1 |
|  | Janajati | 5,428 | 36.6 |
|  | Madhesi | 2,333 | 15.7 |
|  | Muslim | 676 | 4.6 |
|  | Other | 15 | 0.1 |
| Wealth Index | Middle | 3,028 | 20.4 |
|  | Poorer | 2,857 | 19.2 |
|  | Poorest | 2,628 | 17.7 |
|  | Richer | 3,197 | 21.5 |
|  | Richest | 3,135 | 21.1 |
