# Supplementary material for: Spatial heterogeneity of menstrual discriminatory practices against Nepalese women: A population-based study using the 2022 Demographic and Health Survey
Source: PLOS Glob Public Health. 2024 Nov 13;4(11):e0003145. doi: 10.1371/journal.pgph.0003145 (PMC11560001; doi:10.1371/journal.pgph.0003145)
Supplement: S3 Table — (DOCX) [file pgph.0003145.s003.docx]

**S3 Table**: Univariable Poisson regression of menstrual restrictions by social-demographic and economic factors

|  | **Model1: Any restriction** | | | **Model2: Religious restrictions** | | | **Model3: Household level restrictions** | | | **Model4: Chhaupadi** | | |
| --- | --- | --- | --- | --- | --- | --- | --- | --- | --- | --- | --- | --- |
| **Characteristic** | **PR** | **95% CI** | **P-value** | **PR** | **95% CI** | **P-value** | **PR** | **95% CI** | **P-value** | **PR** | **95% CI** | **P-value** |
| **Age (ref: 15-19 years)** | | | | | | | | | | | | |
| 20-24 | 1.01 | 0.98-1.04 | 0.424 | 1.02 | 0.98-1.05 | 0.323 | 0.90 | 0.83-0.97 | 0.008 | 0.79 | 0.64-0.97 | 0.023 |
| 25-29 | 1.01 | 0.98-1.05 | 0.391 | 1.02 | 0.98-1.06 | 0.337 | 0.89 | 0.82-0.98 | 0.017 | 0.66 | 0.53-0.83 | <0.001 |
| 30-34 | 1.04 | 1.01-1.07 | 0.023 | 1.05 | 1.01-1.08 | 0.016 | 0.97 | 0.89-1.06 | 0.546 | 0.76 | 0.62-0.95 | 0.015 |
| 35-39 | 1.04 | 1-1.07 | 0.032 | 1.05 | 1.01-1.09 | 0.009 | 1.02 | 0.94-1.11 | 0.686 | 0.77 | 0.61-0.96 | 0.019 |
| 40-44 | 1.06 | 1.03-1.09 | <0.001 | 1.07 | 1.03-1.1 | <0.001 | 1.02 | 0.93-1.11 | 0.657 | 1.00 | 0.8-1.27 | 0.971 |
| 45-49 | 1.09 | 1.05-1.13 | <0.001 | 1.08 | 1.03-1.12 | 0.001 | 1.16 | 1.06-1.28 | 0.002 | 0.97 | 0.75-1.26 | 0.83 |
| **Highest level of education (ref: No education)** | | | | | | | | | | | | |
| Basic | 0.98 | 0.95-1.01 | 0.153 | 0.98 | 0.95-1.01 | 0.122 | 1.18 | 1.07-1.29 | 0.001 | 0.75 | 0.63-0.9 | 0.002 |
| Secondary | 1.01 | 0.98-1.04 | 0.61 | 1.01 | 0.98-1.04 | 0.484 | 1.35 | 1.22-1.5 | <0.001 | 0.69 | 0.58-0.84 | <0.001 |
| Higher | 1.08 | 1.04-1.12 | <0.001 | 1.11 | 1.06-1.16 | <0.001 | 1.64 | 1.43-1.9 | <0.001 | 0.37 | 0.21-0.66 | 0.001 |
| **Caste/Ethnicity (ref: Janajati)** | | | | | | | | | | | | |
| Brahmin/Chhetri | 1.26 | 1.22-1.31 | <0.001 | 1.19 | 1.15-1.24 | <0.001 | 3.82 | 3.4-4.28 | <0.001 | 8.47 | 5.87-12.22 | <0.001 |
| Dalit | 1.08 | 1.03-1.13 | 0.001 | 1.03 | 0.97-1.08 | 0.342 | 1.97 | 1.69-2.3 | <0.001 | 5.74 | 3.91-8.42 | <0.001 |
| Madhesi | 1.20 | 1.15-1.26 | <0.001 | 1.24 | 1.18-1.29 | <0.001 | 1.36 | 1.08-1.7 | 0.009 | 4.17 | 2.61-6.68 | <0.001 |
| Muslim | 0.96 | 0.84-1.11 | 0.598 | 0.97 | 0.83-1.13 | 0.718 | 0.42 | 0.25-0.7 | 0.001 | 0.00 | 0-0 | <0.001 |
| other | 1.13 | 0.88-1.46 | 0.33 | 1.18 | 0.92-1.52 | 0.198 | 2.93 | 1.53-5.6 | 0.001 | 7.60 | 1.73-33.39 | 0.007 |
| **Residence (ref: Rural)** | | | | | | | | | | | | |
| Urban | 1.03 | 0.99-1.07 | 0.127 | 1.06 | 1.02-1.11 | 0.009 | 0.95 | 0.83-1.08 | 0.423 | 0.53 | 0.39-0.71 | <0.001 |
| **Region/Province (ref: Bagmati)** | | | | | | | | | | | | |
| Gandaki | 1.02 | 0.94-1.1 | 0.682 | 0.98 | 0.89-1.08 | 0.678 | 1.09 | 0.88-1.34 | 0.421 | 1.94 | 1.07-3.51 | 0.029 |
| Karnali | 1.16 | 1.1-1.23 | <0.001 | 1.11 | 1.03-1.18 | 0.003 | 1.89 | 1.63-2.19 | <0.001 | 14.07 | 8.71-22.73 | <0.001 |
| Koshi | 1.09 | 1.03-1.16 | 0.004 | 1.11 | 1.03-1.18 | 0.003 | 1.14 | 0.95-1.37 | 0.173 | 1.77 | 0.93-3.38 | 0.082 |
| Lumbini | 1.06 | 1-1.13 | 0.05 | 1.05 | 0.99-1.13 | 0.121 | 1.10 | 0.91-1.32 | 0.333 | 2.17 | 1.18-3.97 | 0.012 |
| Madhesh | 1.09 | 1.02-1.16 | 0.008 | 1.13 | 1.06-1.21 | <0.001 | 0.44 | 0.34-0.57 | <0.001 | 2.55 | 1.48-4.37 | 0.001 |
| Sudurpashchim | 1.21 | 1.15-1.27 | <0.001 | 1.04 | 0.97-1.12 | 0.224 | 1.99 | 1.67-2.37 | <0.001 | 10.55 | 6.33-17.61 | <0.001 |
| **Gender of the head of the household (ref: Male)** | | | | | | | | | | | | |
| Female | 0.97 | 0.95-1 | 0.02 | 0.98 | 0.95-1 | 0.059 | 0.87 | 0.81-0.94 | <0.001 | 1.12 | 0.95-1.32 | 0.196 |
| **Age of the head of the household (ref: 15-24 years)** | | | | | | | | | | | | |
| 25-34 | 1.04 | 0.98-1.09 | 0.198 | 1.04 | 0.98-1.1 | 0.205 | 1.17 | 0.98-1.4 | 0.089 | 0.98 | 0.68-1.42 | 0.934 |
| 35-44 | 1.05 | 0.99-1.11 | 0.112 | 1.05 | 0.99-1.11 | 0.118 | 1.43 | 1.2-1.71 | <0.001 | 1.18 | 0.81-1.73 | 0.388 |
| 45-54 | 1.07 | 1.01-1.13 | 0.017 | 1.07 | 1.01-1.14 | 0.028 | 1.51 | 1.26-1.81 | <0.001 | 1.10 | 0.75-1.62 | 0.617 |
| 55-64 | 1.09 | 1.03-1.16 | 0.002 | 1.08 | 1.02-1.15 | 0.013 | 1.66 | 1.39-1.99 | <0.001 | 1.08 | 0.74-1.58 | 0.693 |
| >=65 | 1.10 | 1.04-1.17 | 0.001 | 1.07 | 1-1.14 | 0.055 | 1.89 | 1.57-2.26 | <0.001 | 1.34 | 0.91-1.98 | 0.141 |
| **Wealth Index (ref: Poorest)** | | | | | | | | | | | | |
| Middle | 1.02 | 0.98-1.07 | 0.333 | 1.14 | 1.08-1.2 | <0.001 | 0.66 | 0.58-0.76 | <0.001 | 0.40 | 0.29-0.55 | <0.001 |
| Poorer | 1.04 | 0.99-1.08 | 0.104 | 1.13 | 1.07-1.19 | <0.001 | 0.71 | 0.63-0.8 | <0.001 | 0.44 | 0.33-0.58 | <0.001 |
| Richer | 1.04 | 0.99-1.09 | 0.107 | 1.15 | 1.09-1.22 | <0.001 | 0.69 | 0.6-0.8 | <0.001 | 0.28 | 0.2-0.4 | <0.001 |
| Richest | 1.06 | 1.01-1.12 | 0.018 | 1.19 | 1.13-1.26 | <0.001 | 0.87 | 0.76-1 | 0.044 | 0.13 | 0.08-0.2 | <0.001 |
