# Supplementary material for: Spatial heterogeneity of menstrual discriminatory practices against Nepalese women: A population-based study using the 2022 Demographic and Health Survey
Source: PLOS Glob Public Health. 2024 Nov 13;4(11):e0003145. doi: 10.1371/journal.pgph.0003145 (PMC11560001; doi:10.1371/journal.pgph.0003145)
Supplement: S4 Table — (DOCX) [file pgph.0003145.s004.docx]

**S4 Table:** Model goodness-of-fit

| **Model** | **Adjusted** $\boldsymbol{R}^{\mathbf{2}}$ | **AIC** | **Null deviance** | **Complex model deviance** | **P value** |
| --- | --- | --- | --- | --- | --- |
| Model1 | 0.04 | 25695.54 | 3644 | 3493 | <0.001 |
| Model2 | 0.03 | 25442.38 | 4700 | 4551 | <0.001 |
| Model3 | 0.24 | 17594.25 | 9587 | 7232 | <0.001 |
| Model4 | 0.23 | 5083.82 | 4491 | 3430 | <0.001 |
