# Supplementary material for: Spatial heterogeneity of menstrual discriminatory practices against Nepalese women: A population-based study using the 2022 Demographic and Health Survey
Source: PLOS Glob Public Health. 2024 Nov 13;4(11):e0003145. doi: 10.1371/journal.pgph.0003145 (PMC11560001; doi:10.1371/journal.pgph.0003145)
Supplement: Attachment — Submitted filename: Response to Reviewers.docx [file pgph.0003145.s005.docx]

**Spatial heterogeneity of menstrual discriminatory practices against Nepalese women: a population-based study using the 2022 Demographic and Health Survey**

Reviewer Comments:

**Reviewer 1:**

-     While the abstract mentions that menstrual restrictions affect the SDGs, this discussion is not reflected in the introduction. Consider integrating this aspect into the introduction.

**Response:** We appreciate the reviewer’s suggestion. We have included the aspect of SGDs in the introduction. **Line 59-65** as shown below:

Menstrual health is an important public health and human rights challenge, influencing physical and mental well-being, gender equality, and socioeconomic participation [1]. Inadequate menstrual health management contributes to health disparities, educational barriers, and infringes on dignity and equal human rights [2]. Despite the clear evidence, menstrual health and hygiene continue to pose significant public health and social challenges in Low- and Middle-Income Countries (LMIC)[2–5]. Therefore, addressing menstrual health barriers remains essential for achieving the United Nations Sustainable Development Goals (SDGs), particularly those related to health, education, gender equality, and reducing inequalities[6].

-     Motivate the importance of menstrual health by framing it as a public health and human rights issue, drawing on literature such as Babbar et al. (2022) and Hennegan (2017).

**Response:** We appreciate the reviewer's feedback. We have added this suggestion in the introduction section. **Line 59-65** as shown below:

Menstrual health is an important public health and human rights challenge, influencing physical and mental well-being, gender equality, and socioeconomic participation [1]. Inadequate menstrual health management contributes to health disparities, educational barriers, and infringes on dignity and equal human rights [2]. Despite the clear evidence, menstrual health and hygiene continue to pose significant public health and social challenges in Low- and Middle-Income Countries (LMIC)[2–5]. Therefore, addressing menstrual health barriers remains essential for achieving the United Nations Sustainable Development Goals (SDGs), particularly those related to health, education, gender equality, and reducing inequalities[6].

-     Given that all four authors are from Kenya and are working with data from Nepal, it would be beneficial for the authors to provide justification for the use of Nepalese data, demonstrating their understanding of the background and context.

**Response:** We appreciate the reviewer's feedback and would like to clarify our rationale for using Nepalese data in our study. The 2022 Nepal Demographic and Health Survey (DHS) is uniquely comprehensive, offering the most recent and detailed dataset on menstrual discrimination linked to Geographic Information System (GIS) data. This integration allows for an in-depth analysis of geographic micro-variations in menstrual discriminatory practices, which is central to our research. During our extensive literature review, we identified significant gaps in the current understanding of menstrual discrimination, particularly regarding its spatial distribution and impact. The Nepal 2022 DHS dataset fills these gaps, providing critical insights that feed into our ongoing systematic review and meta-analysis study of menstrual discrimination in Africa and Asia. This broader research aims to develop important recommendations on the utility of spatial analytics and targeted interventions, thus enhancing global efforts to combat menstrual discrimination.

**Abstract**
-     Clarify the sample size in the abstract.

**Response:** We thank the reviewer for this comment, we have included a statement on the sample size: **Lines 36-37**

“The analysis was based on data from the 2022 Nepal Demographic and Health Survey which included 13,065 women aged 15–49 who reported menstruating within the past year.”

-    In the results section of the abstract, include corresponding beta coefficients and p-values for clarity.

Response: We thank the reviewer for this comment, we have included the beta coefficients and p-values. **Lines 46-50**

“Geographical variations were more prominent for women experiencing Chhaupadi (primary geographical cluster: relative risk=7.4, p<0.001). Strikingly, women who reside in households led by female household heads were less likely to report experiencing household-level restriction during menstruation (Adjusted prevalence ratio (aPR) =0.89, [95%CI: 0.84–0.94],p<0.001) whilst those

residing in wealthy households were less likely to report experiencing Chhaupadi (aPR =0.26, [95%CI: 0.17-0.39], p<0.001; among the richest).”

-  The literature around menstrual health in Nepal appears limited, as evidenced by the reliance on global literature in the introduction. Consider citing literature from neighboring countries like India and Bangladesh, which share similar socio-economic contexts.

**Response**: We thank the reviewer for this comment, we have enhanced our literature with additional citations covering a number of Asian countries affected by menstrual stigma: **Line 66-68.**

Across LMIC, millions of women continue to encounter difficulties in managing their menstrual periods while preserving their dignity. This is predominantly attributed to period poverty[7–12], internalized stigma, shame rooted in myths and misconceptions surrounding menstruation [2,13–18].

**Lines: 72-75**

In the South Asian context, Nepal is one of the most affected countries, with over 90% of menstruating girls and women experiencing some form of discriminatory practices during their menstrual periods[19]. These practices are spread across many South Asian countries where menstruation is considered taboo [20–24], though with varying degrees of intensity.

-  Provide a table explaining how control variables are created for clarity

**Response**: We thank the reviewer for this comment. We have provided a table below detailing the various control variables and how they were constructed. **Line 414**

**Supp. Table 1**: Factors associated with menstrual taboos/restrictions

| **Predictor/**  **control variable** | **Variable type as recorded in 2022 NDHS** | **Level of Data used in deriving the variable** | **Variable used in the study** | **Categories/**  **groups** |
| --- | --- | --- | --- | --- |
| Age | Continuous and Categorical | Individual level data | Age in years of the respondent categorized into 7 groups. | 15-19, 20-24, 25-29, 30-34, 35-39, 40-44, 45-49 |
| Education level | Categorical | Individual level data | Highest level of education of the survey respondent categorized into 4 groups | None, Basic, Secondary, Higher |
| Caste/Ethnicity | Categorical | Individual level data | Respondent’s ethnicity grouped into 6 categories. Janajati (hill janajati, terai janajati, newar), Madhesi (terai brahmin/chhetri, other terai caste), Dalit(hill dalit, terai dalit), Brahmin/Chhetri(hill Brahmin, hill chhetri), Muslim, Others | Janajati, Madhesi, Dalit, Brahmin/Chhetri,  Muslim, Others. |
| Region | Categorical | Individual level data | The seven first level administrative regions (provinces) of Nepal where the respondent resided at the time of the survey. | Bagmati, Gandaki, Karnali, Koshi, Lumbini, Madhesh, Sudurpashchim |
| Gender of the household head | Categorical | Individual level data | Gender of the household head as reported by the respondent. | Male, Female |
| Age of the household head | Continuous | Individual level data | Age of the household head as reported by the respondent. It was categorized into 6 groups | 15-24, 25-34, 35-44, 45-54, 55-64, 65 and over |
| Residence | Categorical | Individual level data | Current area of residence whether urban(metropolitan, sub-metropolitan or municipality) or rural | Rural, urban |
| Wealth index | Categorical | Household data | In DHS surveys, household characteristics used to assess wealth are assigned factor scores using the principal components analysis (PCA). Using the first principal component, the sample is divided into quintiles, each with an equal number of households. Key characteristics included in this analysis were: drinking water source, toilet type, shared facilities, materials for the floor, walls, and roof, cooking fuel, household services, and possessions such as electricity, TV, vehicles, land size, livestock, bank accounts, and window types. | Poorest, poorer, middle, richer, richest. |

-  Explain the Taylor linearization approach used to account for the complex sampling design and clustering.

Response: We thank the reviewer for this comment. Taylor linearization is essential for accurate standard error estimation in complex survey data, as it addresses design effects from clustering and stratification. Unlike simple random sampling, complex surveys involve multiple stages and varied selection probabilities, leading to correlated observations. Taylor linearization approximates variance using a first-order expansion, capturing these design effects effectively. This method yields more reliable standard errors that better reflect the true variability. We have included a citation to provide more details as shown in line **166-168**.

“The prevalence of menstrual restrictions by social demographic characteristics were estimated accounting for the study design. To account for the complex sampling design, the Taylor linearization approach was used to calculate the standard errors [26,27].”

- Clarify the rationale behind creating risk maps and conducting poison regression. It's essential to explain to readers why these methods are chosen and how they contribute to the analysis, rather than simply stating their use.

**Response**: We thank the reviewer for this important comment. We have clarified the rationale behind risk maps in the manuscript. **Lines 96-103.**

“Previous studies indicate regional distribution patterns across Nepal with particularly high prevalence estimates in Karnali and Sudurpashchim provinces [19,28,29]. However, these estimates mainly provide provincial-level estimates, excluding fine-scale variation that may exist within provinces. Therefore, the development of fine-scale risk stratification maps of menstrual discriminatory practices means that the most vulnerable populations can be identified more accurately and prioritized with scarce resources. In addition, the risk maps can aid the establishment of spatial patterns of menstrual exclusions for contextualizing changes in the burden of menstrual stigma relative to interventional policies and programs.”

- The modified Poisson regression model is widely used for binary outcomes when risk ratios are desired. Unlike the log-binomial model which has convergence issues, the modified Poisson regression model has been shown to provide unbiased estimates when the study design is appropriate. In addition, the risk factors are easier to interpret compared to odds ratios. We have added the following statements in **line 193-194** of the manuscript and provided relevant citations:

“Modified Poisson regression with robust standard errors [30,31] was used to assess the factors influencing heterogeneity in menstrual restriction outcomes.”

______________________________________________________________________________

**Reviewer 2:**

1. Methodology.
a) Data Collection: Information on how menstrual restrictions were measured and assessed within the survey should be provided. This would help readers understand the reliability and validity of the data used for analysis.

**Response:** We thank the reviewer for raising this important question. We have summarized this in the manuscript and provided a citation to the main document for further detail as follows: **Line 146-157**

“Our study sought to describe menstrual discriminatory practices among Nepalese women. These practices include restrictions on activities such as entering the temple, participating in religious activities, cooking, handling food items, fetching water, eating with the family, and the change in sleeping arrangements within the household during menstruation. In the 2022 NDHS, all eligible survey participants were asked whether or not were excluded from participating in these activities during menstruation (Yes/No). In our analyses, menstrual restriction activities were grouped into four binary composite outcomes: a) any form of menstrual restriction defined as any menstrual-related restriction, b) religious restrictions defined as exclusions from entering the temple or restrictions on participating in religious activities, c) household level restriction defined as restrictions from fetching water, cooking and handling food items, eating with the family, sleeping in the main house, and sleeping with the husband, and d) Chhaupadi defined as exclusion from sleeping in the main house as described previously [32]. For each composite binary outcome, {1} indicates an experience of at least one restriction within the defined sub-group and {0} otherwise.”

b) Statistical Analysis: While the study employed appropriate statistical methods such as Poisson regression and spatial scan statistics, additional information on model assumptions, goodness-of-fit tests, and potential confounders should be included to strengthen the analysis

**Response:** We thank the reviewer for this comment. Additional information on model assumptions has been included as supplemental material in the manuscript.

**Line 194-195**.

“The generalized variance inflation factors VIF were calculated to test for potential multicollinearity.”

- The DHS capture variables as categorical and thus the **linearity assumption** was not applicable

Parameters on model goodness of fit have been included in **Supp. Table 4** of the supplementary material. **Line 421**

**Supp. Table 4 : Model goodness-of-fit.**

| **Model** | **Adjusted** $\boldsymbol{R}^{\mathbf{2}}$ | **AIC** | **Null deviance** | **Complex model deviance** | **P value** |
| --- | --- | --- | --- | --- | --- |
| Model1 | 0.04 | 25695.54 | 3644 | 3493 | <0.001 |
| Model2 | 0.03 | 25442.38 | 4700 | 4551 | <0.001 |
| Model3 | 0.24 | 17594.25 | 9587 | 7232 | <0.001 |
| Model4 | 0.23 | 5083.82 | 4491 | 3430 | <0.001 |

2. **Results**.
a) **Interpretation of Findings**: The authors present prevalence rates for different forms of menstrual restrictions across regions but do not discuss potential reasons for these variations or compare them with previous studies. Providing a contextual interpretation of these findings would enhance their significance.

**Response:** We thank the reviewer for raising this important question. The spatial disparity in menstrual exclusions can be partially attributed to variations in ethnic compositions. Higher prevalence rates are noted in areas predominantly inhabited by Brahmin and Chhetri communities, where such practices are common. In these communities, religious leaders often play a key role in enforcing these practices. We have cited relevant studies that support these observations and provide further context for these findings. However, our findings highlight a large fraction of unexplained variability suggesting presence of unmeasured variables that require further research.

Detailed information is provided in lines: **Line 273-276**

“Menstrual impurity is a concept rooted in Hinduism, with religious leaders often upholding this practice [33]. The Brahmins, who frequently serve as priests, along with high-caste Hindu women like Chhetris, are often expected to observe and enforce these rituals [34].”

b) **Statistical Analysis:** While the study employed appropriate statistical methods such Spatial Analysis: The study utilizes kernel density estimation and local cluster detection techniques to identify high-prevalence clusters of menstrual restrictions. However, it is unclear how these clusters were defined or what criteria were used to determine their significance.

**Response:** We thank the reviewer for raising this question. We have clarified this in the methods sections of the manuscript and provided a clearer definition of a cluster as follows: **line 170-187**

“The scan statistic compares case counts inside and outside each circle, generating an observed log-likelihood statistic that is evaluated against the null hypothesis of complete spatial randomness. In this study, Monte-Carlo simulation with 999 replications was used to create permutations of the observed cases across all data locations generating the simulated log-likelihoods. The observed log-likelihood and the simulated log-likelihoods are used to calculate the p-value for statistical significance testing. The Gini coefficient determined optimal non-overlapping cluster sizes. Hereinafter, a cluster is defined as a geographical area experiencing significantly (p-value <0.05) higher prevalence of menstrual discrimination than the average.”

3. Discussion.
a) **Limitations**: The authors acknowledge limitations related to self-reported data and social desirability bias but do not discuss other potential sources of bias or limitations inherent in cross-sectional surveys.

**Response:** We thank the reviewer for raising this point. Our cross-sectional study design cannot establish causality because data is collected at a single time point. In addition, our study design may be subject to selection and recall bias. We have clarified this in the methods sections of the manuscript as follows: **lines 346-348.**

“In addition, our analyses relied on reported cross-sectional data that may be subject to social desirability and recall bias and cannot establish causality. However, our regional estimates of prevalences are consistent with previous quantitative studies [19,29,35] suggesting that the bias is likely minimal.”

b) **Generalizability**: While the study focuses on Nepal, it does not discuss whether its findings can be extrapolated to other countries or regions facing similar challenges with menstrual discrimination.

**Response:** We thank the reviewer for this comment. We have clarified this in the methods sections of the manuscript as follows: **lines 348-351.**

“Lastly, our study was conducted in a country experiencing high prevalences of menstrual discrimination resulting from deeply rooted religious and cultural norms. Therefore, our findings can only be generalized to regions with similar religious and cultural contexts and may not apply to other settings.”

4. **Policy implications**.
a) **Policy recommendation**: The study briefly mentions targeted interventions but does not provide specific recommendations for policymakers or practitioners working towards eliminating menstrual discriminatory practices in Nepal.

**Response:** We thank the reviewer for raising this important question. We have also added the following text in **line 52 -57**

“Our study demonstrated marked geographical micro-variations in menstrual discriminatory practices in Nepal. Policymakers should implement preventive behavioral interventions in the most vulnerable geographic areas to effectively and efficiently reduce the overall prevalence of menstrual discrimination. It is crucial to prioritize designing and testing targeted interventions to determine their effectiveness against Chhaupadi in these high-prevalence settings. Additionally, empowering women appears to be a promising strategy in combating menstrual discrimination within the household.”

We have also added the following text in **line 360-365**

“Given that Chhaupadi is deeply ingrained in complex cultural and religious contexts, our study recommends strategies targeting key enforcers, such as religious leaders, mothers, heads, and older family members, who play critical roles in perpetuating menstrual taboos and rituals. These policy interventions may include a combination of women empowerment, advocacy, and educational awareness of the current laws illegalizing menstrual discrimination, and associated health risks[33,35,36].”

b) **Future research directions**: It would be beneficial if the authors suggest areas for future research based on their findings, such as exploring cultural norms surrounding menstruation or evaluating existing interventions to reduce menstrual restrictions.

**Response:** Thank you for your valuable feedback on our manuscript. We have added the following text to line **327 -333**

“Despite the robust statistical approaches used in this study, a substantial portion of the variability in the regression models remained unexplained. Therefore, there is a need for additional research to identify the unmeasured determinants of menstrual discrimination that may act at various geographic scales. Understanding these unmeasured predictor variables may enhance the development of interventions that address the root cause of menstrual discrimination. Interventions designed against menstrual discriminatory practices should not only target high-prevalence clusters but also incorporate a research component to test their utility and cost-effectiveness in real-world settings.”

**References**

1.	Hennegan J, Winkler IT, Bobel C, Keiser D, Hampton J, Larsson G, Chandra-Mouli V, Plesons M, Mahon T. Menstrual health: a definition for policy, practice, and research. Sex Reprod Health Matters. 29(1):1911618.

2.	Babbar K, Martin J, Ruiz J, Parray AA, Sommer M. Menstrual health is a public health and human rights issue. The Lancet Public Health. 2022 Jan 1;7(1):e10–1.

3.	Hennegan J, Shannon AK, Rubli J, Schwab KJ, Melendez-Torres GJ. Women’s and girls’ experiences of menstruation in low- and middle-income countries: A systematic review and qualitative metasynthesis. PLOS Medicine. 2019 May 16;16(5):e1002803.

4.	Krenz A, Strulik H. The impact of menstruation hygiene management on work absenteeism of women in Burkina Faso. Economics & Human Biology. 2021;43:101067.

5.	Kuhlmann AS, Henry K, Wall LL. Menstrual Hygiene Management in Resource-Poor Countries. Obstet Gynecol Surv. 2017 Jun;72(6):356–76.

6.	Sommer M, Torondel B, Hennegan J, Phillips-Howard PA, Mahon T, Motivans A, Zulaika G, Gruer C, Haver J, Caruso BA, Monitoring Menstrual Health and Hygiene Group. How addressing menstrual health and hygiene may enable progress across the Sustainable Development Goals. Global Health Action. 2021 Jan 1;14(1):1920315.

7.	Babbar K, Saluja D, Sivakami M. How socio-demographic and mass media factors affect sanitary item usage among women in rural and urban India. Waterlines. 2021;(3):160–78.

8.	Babbar K, Garikipati S. What socio-demographic factors support disposable vs. sustainable menstrual choices? Evidence from India’s National Family Health Survey-5. PLOS ONE. 2023 Aug 17;18(8):e0290350.

9.	Chakrabarty M, Singh A, Let S, Singh S. Decomposing the rural–urban gap in hygienic material use during menstruation among adolescent women in India. Scientific Reports. 2023;13(1):22427.

10.	Singh A, Chakrabarty M. Spatial heterogeneity in the exclusive use of hygienic materials during menstruation among women in urban India. PeerJ. 2023 Mar 20;11:e15026.

11.	Goli S, Sharif N, Paul S, Salve PS. Geographical disparity and socio-demographic correlates of menstrual absorbent use in India: A cross-sectional study of girls aged 15–24 years. Children and Youth Services Review. 2020 Oct 1;117:105283.

12.	Alugnoa DN, Cousins T, Sato M. Period poverty and menstrual belonging: a matter of climate justice. The Lancet Planetary Health. 2022 Jul 1;6(7):e551–2.

13.	UNICEF U. Menstrual hygiene | UNICEF [Internet]. Menstrual hygiene. 2022 [cited 2023 Dec 14]. Available from: https://www.unicef.org/wash/menstrual-hygiene

14.	ActionAid. Chhaupadi and menstruation taboos | ActionAid UK [Internet]. 2024 [cited 2024 Feb 12]. Available from: https://www.actionaid.org.uk/our-work/period-poverty/chhaupadi-and-menstruation-taboos

15.	Health TLC& A. Normalising menstruation, empowering girls. The Lancet Child & Adolescent Health. 2018 Jun 1;2(6):379.

16.	Health–Americas TLR. Menstrual health: a neglected public health problem. Lancet Regional Health-Americas [Internet]. 2022 [cited 2024 Feb 11];15. Available from: https://www.ncbi.nlm.nih.gov/pmc/articles/PMC9903918/

17.	Lonkhuijzen VRM, Garcia FK, Wagemakers A. The Stigma Surrounding Menstruation: Attitudes and Practices Regarding Menstruation and Sexual Activity During Menstruation. Women’s Reproductive Health. 2023 Jul 3;10(3):364–84.

18.	UN. International Women’s Day - 8 March 2019Women’s menstrual health should no longer be a taboo [Internet]. OHCHR. 2019 [cited 2023 Dec 14]. Available from: https://www.ohchr.org/en/press-releases/2019/03/international-womens-day-8-march-2019womens-menstrual-health-should-no

19.	UNICEF. Multiple Indicator Cluster Surveys Final Report 2019 | UNICEF Nepal [Internet]. 2020 [cited 2024 Feb 12]. Available from: https://www.unicef.org/nepal/reports/multiple-indicator-cluster-survey-final-report-2019

20.	Alam MU, Luby SP, Halder AK, Islam K, Opel A, Shoab AK, Ghosh PK, Rahman M, Mahon T, Unicomb L. Menstrual hygiene management among Bangladeshi adolescent schoolgirls and risk factors affecting school absence: results from a cross-sectional survey. BMJ Open. 2017 Jul 1;7(7):e015508.

21.	Garg S, Anand T. Menstruation related myths in India: strategies for combating it. J Family Med Prim Care. 2015;4(2):184–6.

22.	Mahon T, Fernandes M. Menstrual hygiene in South Asia: a neglected issue for WASH (water, sanitation and hygiene) programmes. Gender & Development. 2010 Mar 1;18(1):99–113.

23.	Priandhita R, Lahiri-Dutt K. The power of the blood: myths and practices surrounding menstruation in Indonesian diamond mining. South East Asia Research. 2023 Jan 2;31(1):72–86.

24.	Rothchild J, Piya PS. Rituals, Taboos, and Seclusion: Life Stories of Women Navigating Culture and Pushing for Change in Nepal. In: Bobel C, Winkler IT, Fahs B, Hasson KA, Kissling EA, Roberts TA, editors. The Palgrave Handbook of Critical Menstruation Studies [Internet]. Singapore: Springer; 2020 [cited 2023 Nov 8]. p. 915–29. Available from: https://doi.org/10.1007/978-981-15-0614-7_66

25.	McCammon E, Bansal S, Hebert LE, Yan S, Menendez A, Gilliam M. Exploring young women’s menstruation-related challenges in Uttar Pradesh, India, using the socio-ecological framework. Sex Reprod Health Matters. 28(1):1749342.

26.	Seidenberg AB, Moser RP, West BT. Preferred Reporting Items for Complex Sample Survey Analysis (PRICSSA). Journal of Survey Statistics and Methodology. 2023 Sep 1;11(4):743–57.

27.	Lumley T. Complex surveys: a guide to analysis using R [Internet]. John Wiley & Sons; 2011

28.	Crawford M, Menger LM, Kaufman MR. ‘This is a natural process’: managing menstrual stigma in Nepal. Culture, Health & Sexuality. 2014 Apr 21;16(4):426–39.

29.	Amatya P, Ghimire S, Callahan KE, Baral BK, Poudel KC. Practice and lived experience of menstrual exiles (Chhaupadi) among adolescent girls in far-western Nepal. Dreibelbis R, editor. PLoS ONE. 2018 Dec 10;13(12):e0208260.

30.	Yelland LN, Salter AB, Ryan P. Performance of the modified Poisson regression approach for estimating relative risks from clustered prospective data. American journal of epidemiology. 2011;174(8):984–92.

31.	Zou G. A modified poisson regression approach to prospective studies with binary data. American journal of epidemiology. 2004;159(7):702–6.

32.	ICF NM of H and P New ERA and. Nepal Demographic and Health Survey 2022. 2023 Jun 15 [cited 2023 Nov 11]; Available from: https://dhsprogram.com/publications/publication-FR379-DHS-Final-Reports.cfm

33.	Amery F, Channon M, Puri MC, Thomson J. Developing the menstrual justice agenda: insights from a mixed method study in the mid-western region of Nepal. Sexual and Reproductive Health Matters. 2023 Dec 31;31(1):2204025.

34.	HighBeam. Brahman and Chhetri of Nepal | Encyclopedia.com [Internet]. 2024 [cited 2024 Aug 10]. Available from: https://www.encyclopedia.com/humanities/encyclopedias-almanacs-transcripts-and-maps/brahman-and-chhetri-nepal

35.	Thakuri DS, Thapa RK, Singh S, Khanal GN, Khatri RB. A harmful religio-cultural practice (Chhaupadi) during menstruation among adolescent girls in Nepal: Prevalence and policies for eradication. Federici S, editor. PLoS ONE. 2021 Sep 1;16(9):e0256968.

36.	Thapa S, Aro AR. “Menstruation means impurity”: multilevel interventions are needed to break the menstrual taboo in Nepal. BMC Womens Health. 2021 Feb 28;21(1):84.
